# Understanding the pathways to workplace flourishing: the effect of trust and psychological safety in high-pressure work environments

**DOI:** 10.3389/fpsyt.2026.1912314

**Published:** 2026-08-27

**Authors:** Saviour Ayertey Nubuor, Theresa Esi Bosomtwe, Rosemond Obuobi, Harry Siaw Bampoe, Diana Efua Bosomtwe-Duker

**Affiliations:** 1 Department of Organisation and Human Resource Management, University of Ghana Business School, University of Ghana, Accra, Ghana; 2 Department of Human Resource Management, Knutsford University, Accra, Ghana; 3 Department of Management and Public Administration, Accra Technical University, Accra, Ghana; 4 Department of Marketing and Management studies, Wisconsin International University College, Accra, Ghana; 5 37Military Nursing and Midwifery Training College -37NMTC, Accra, Ghana

**Keywords:** employee well-being, Ghana armed forces, hierarchical organizations, job demands-resources theory, military personnel, occupational health, psychological safety, trust in leadership

## Abstract

**Introduction:**

Drawing on the Job Demands-Resources (JD-R) theory, this study examines the associations between psychological safety, trust in leadership, and workplace flourishing within the high-pressure, hierarchical context of the Ghana Armed Forces.

**Methods:**

Using a cross-sectional quantitative research design, data were collected from 292 military personnel stationed at a single military hospital through stratified sampling (response rate = 78.9%). Structural Equation Modeling (SEM) with bootstrapped indirect effects was employed to test the hypothesized model, with Confirmatory Factor Analysis confirming construct validity.

**Results:**

The results reveal significant direct associations between trust in leadership (β = .296, p <.001) and psychological safety (β = .111, p <.05) with workplace flourishing. Critically, serial mediation analysis revealed that psychological safety is indirectly associated with workplace flourishing through trust in leadership (Indirect Effect = .0781, 95% CI [.0137,.0939]), suggesting that in hierarchical military contexts, psychological safety function as a climate resource that enables trust development, which in turn is associated with flourishing. While trust demonstrates a stronger statistical association, this does not imply causal precedence.

**Discussion:**

The findings extend the JD-R framework to military settings by demonstrating the sequential resource pathway from psychological safety through trust to flourishing at work. Organizations should prioritize creating psychologically safe environments first, as this appears to enable trust development, which then contributes to flourishing. The study offers insights for leadership development in high-stakes environments, while acknowledging that the cross-sectional and self-reported design precludes causal conclusions and limits generalizability.

## 1 Introduction

In contemporary organizational scholarship, the pursuit of employee well-being has evolved beyond a peripheral concern to a central determinant of sustainable performance. While task execution remains vital, a growing body of evidence underscores that long-term organizational resilience and innovation are rooted in a workforce that does more than just function—it thrives (1, 2). This state of optimal well-being, termed workplace flourishing, encompasses the holistic experience of feeling good and functioning effectively, integrating high levels of emotional, psychological, and social well-being (3, 4). Flourishing employees exhibit heightened engagement, robust resilience, and a profound sense of purpose, which collectively translate into superior individual and organizational outcomes (5, 6). Consequently, understanding the levers that cultivate such an environment is a critical imperative for leaders across sectors.

Nowhere is this imperative more pressing than in high-stakes, hierarchical environments such as the military. Military personnel operate under conditions characterized by extreme pressure, unambiguous chains of command, and life-or-death consequences (7). While this structure is essential for discipline and operational effectiveness, it can simultaneously create a paradox for well-being. The very hierarchy that ensures order may inadvertently suppress the individual autonomy, open communication, and psychological security that are fundamental to flourishing (8). In such contexts, traditional performance metrics are insufficient; the psychological and emotional fortitude of personnel becomes a critical component of mission readiness and national security. Therefore, identifying the mechanisms that enable personnel to flourish despite these structural and operational constraints is of paramount importance. To this end, two constructs have emerged as particularly salient: trust in leadership and psychological safety.

Trust, defined as the willingness to be vulnerable to a leader’s actions based on positive expectations (9), is the bedrock of the leader-follower relationship in high-reliability organizations. Psychological safety, the shared belief that one can speak up with ideas, questions, and concerns without fear of reprisal (10), creates the interpersonal climate necessary for learning and risk-taking. Extant research has independently linked both trust and psychological safety to positive outcomes, including improved performance, innovation, and well-being (11, 12).

While these independent associations are well-documented, a critical gap remains. Specifically, the sequential and directional nature of the relationship between trust and psychological safety in hierarchical contexts is not well understood. Research suggests that the relationship may be bidirectional—trust and psychological safety may mutually reinforce each other over time (13–15). However, existing studies have not systematically tested which direction is more dominant in hierarchical military settings. Does trust in leadership enable psychological safety, or does psychological safety enable trust? This question is theoretically important because the answer has different implications for organizational interventions: if trust precedes psychological safety, leadership development should prioritize trust-building; if psychological safety precedes trust, creating safe environments should be the priority.

The Ghana Armed Forces (GAF) provides a unique context for examining this question. As a tri-service military organization operating in a Sub-Saharan African context, the GAF presents a hierarchical structure rarely studied in organizational psychology research. Most studies on trust, psychological safety, and flourishing have been conducted in Western or Asian civilian contexts (16–18), leaving a significant gap in understanding how these dynamics operate in African military settings. The GAF’s unique combination of rigid hierarchy, operational pressure, and diverse personnel from Army, Navy, and Air Force branches offers an opportunity to test whether established relationships hold in this under-researched context.

Drawing on the Job Demands-Resources (JD-R) theory (19), this study propose that psychological safety may function as a climate resource that enables the development of trust—a relational resource—which in turn contributes to flourishing. This sequential resource pathway suggests that in hierarchical military contexts, personnel may first need to perceive that the environment is psychologically safe before they can develop trust in their leaders. This study tests a serial mediation model examining whether psychological safety is indirectly associated with workplace flourishing through trust in leadership (Psychological Safety → Trust → Workplace Flourishing). However, given the bidirectional evidence in the literature suggesting that trust may also facilitate psychological safety (13, 15, 20), thus, the study also examine the competing reverse direction: whether trust in leadership is indirectly associated with workplace flourishing through psychological safety (Trust → Psychological Safety → Workplace Flourishing). Testing both directions allows us to determine which sequential pathway is more dominant in the hierarchical military context.

By examining these competing pathways, this research aims to make a significant contribution to both theory and practice. Theoretically, it extends the JD-R framework by testing a sequential resource pathway in a military context, clarifying the directional relationship between trust and psychological safety or vice versa. Practically, it provides military leaders with evidence-based insights into whether to prioritize creating psychologically safe environments or building trust directly to enhance personnel flourishing. While the study cross-sectional design precludes causal conclusions, the findings offer preliminary evidence regarding these relationships in a hierarchical military context.

## 2 Literature review

### 2.1 Workplace flourishing

Workplace flourishing represents a pivotal and multi-dimensional construct in organizational psychology, moving beyond the mere absence of illness to capture a state of optimal well-being where employees thrive by both feeling good and functioning effectively (1, 3). It is holistically characterized by the presence of positive emotions and job satisfaction (emotional well-being), a sense of purpose, autonomy, and engagement (psychological well-being), and positive relationships with a sense of social integration and contribution (social well-being) (21, 22). This integrative state is critically important as it equips individuals with the resilience, creativity, and vitality necessary to perform effectively, particularly in demanding environments, while simultaneously driving organizational outcomes such as heightened productivity, reduced turnover, and sustained competitive advantage (23, 24). Consequently, fostering flourishing is not merely a benevolent aim but a strategic imperative for building a resilient and effective workforce, especially within high-stakes contexts like the military where human capability is paramount.

#### 2.1.1 Construct distinctions

It is important to distinguish workplace flourishing from related but distinct constructs frequently examined in organizational research. While flourishing encompasses feeling good and functioning well across emotional, psychological, and social dimensions (1, 3), other constructs capture narrower aspects of well-being:

- Job satisfaction refers to an employee’s evaluative judgment of their job (25) and represents a component of emotional well-being but does not capture psychological or social functioning.
- Affective commitment reflects emotional attachment to and identification with an organization (26) and is related to but distinct from flourishing.
- Work engagement involves vigor, dedication, and absorption in one’s work (27) and corresponds to the psychological well-being dimension of flourishing but does not capture social well-being.
- Subjective well-being encompasses life satisfaction and positive affect (28) and is related to the emotional well-being component of flourishing but does not include functioning dimensions.

In this review, this paper focus on studies that have explicitly measured workplace flourishing using established scales such as the Flourishing at Work Scale (29) or the Mental Health Continuum (30).

#### 2.1.2 Relevance of flourishing dimensions in military contexts

While flourishing encompasses emotional, psychological, and social well-being, these dimensions have differential relevance in military settings. Psychological well-being—characterized by a sense of purpose, mastery, resilience, and work engagement—may be particularly salient in military contexts where personnel face high-stakes operations, prolonged stress, and the need for sustained mental fortitude (4). Military personnel who derive meaning from their service and experience a sense of mastery over their roles may be better equipped to withstand operational pressures. Social well-being—including social acceptance, integration, and contribution—is also critical in military settings where teamwork, unit cohesion, and interdependence are fundamental to mission success (31). In contrast, emotional well-being—while important—may be more variable and influenced by immediate operational conditions (22).

### 2.2 Trust in leadership

Trust is a dynamic psychological state, central to the modern employment relationship, where followers willingly accept vulnerability based on positive expectations of their leader’s character and actions. Contemporary research underscores that this trust is cultivated through a leader’s demonstrable authenticity, ethical transparency, and genuine concern for employee well-being, which fosters a secure and supportive environment (32, 33). This foundation of trust is critical as it directly catalyzes a range of positive organizational outcomes; it not only enhances employee engagement, commitment, and task performance but also serves as a vital foundation for psychological safety—empowering employees to voice ideas and take interpersonal risks without fear (34, 35).

### 2.3 Psychological safety

Psychological safety, rooted in the work of Schein and Bennis (36), is defined as a shared belief that the team or organizational environment is safe for interpersonal risk-taking (10). It is the felt permission to be oneself, to express thoughts and concerns candidly, and to admit mistakes without fear of negative consequences to one’s self-image, status, or career (37, 38). This climate is not about lowering performance standards but about creating an atmosphere of mutual respect and trust where candid dialogue and learning can flourish (39). The consequences of psychological safety are profound and well-documented. It is a critical enabler of performance and innovation, as it allows employees to focus on their work without distraction, share information proactively, and collaborate effectively (40, 41). It also promotes learning behaviors such as seeking feedback, discussing errors, and sharing knowledge (10, 42).

*Level of Analysis Consideration*

While Edmondson’s (10) psychological safety scale was originally designed for team-level assessment, the scale has been widely validated and used at the individual level of analysis in numerous organizational studies (e.g., 12, 41, 43). Individual-level analysis is appropriate for this study for several reasons. First, in hierarchical military contexts, individuals within the same team may experience psychological safety differently based on their rank, position, and relationship with leadership. A junior soldier may perceive less safety than an officer within the same unit, and individual-level analysis captures this variation. Second, psychological safety operates at multiple levels—individual perceptions of team climate, team-level shared beliefs, and organizational-level climate (38).

### 2.4 Relationship between trust and psychological safety

The link between trust in leadership and psychological safety has gained attention as organizations strive to boost employee engagement and performance. Basit (13) studied nurses in Kuala Lumpur and found that when employees trust their supervisors, they feel psychologically safe, which in turn enhances their job engagement. This creates a supportive environment where staff can share their thoughts freely, fostering a sense of obligation to reciprocate that trust and engage more fully in their work. Building on this, Mitterer and Mitterer (14) argue that psychological safety can also lay the groundwork for trust within teams. Their research indicates that when employees feel safe, they are more likely to build trusting relationships with colleagues and managers, which is vital for collaboration and innovation. Mitterer and Mitterer (14) conducted a quantitative study investigating the interplay between psychological safety, trust, and job satisfaction among employees, specifically focusing on a sample of 283 nurses at a large teaching hospital. Their research indicates that when employees feel safe, they are more likely to build trusting relationships with colleagues and managers, which is vital for collaboration and innovation. Hence, authors argue that psychological safety is a crucial foundation for that trust, positing that when employees feel safe, they are more likely to build trusting relationships with their peers and management.

#### 2.4.1 Contradictory and context-dependent findings

However, the relationship between trust and psychological safety is not uniformly positive across all contexts. Research shows that high levels of trust lead to greater psychological safety, which helps reduce turnover intentions (15, 20). This connection is especially strong in hierarchical settings, where trust in leaders becomes critical for employees to feel safe and included (44). Conversely, Maximo, Stander, and Coxen (45) found that trust in supervisors did not significantly mediate the relationship between authentic leadership and psychological safety in a mining context, suggesting that industry and organizational culture may moderate this relationship. Similarly, studies in highly bureaucratic organizations have found weaker associations between trust and psychological safety due to rigid procedural constraints (46). These contradictory findings highlight the need for context-specific investigations, particularly in hierarchical military settings where trust dynamics may operate differently.

#### 2.4.2 Boundary conditions and moderators

The trust-psychological safety relationship is likely moderated by several factors. Organizational culture—particularly the degree of hierarchy and formalization—may strengthen or weaken the relationship. In highly hierarchical contexts like the military, rank differences may moderate how trust translates into psychological safety; junior personnel may require greater trust to feel safe compared to senior personnel. Additionally, leadership style may serve as a boundary condition; transformational and authentic leadership may strengthen the trust-safety relationship, while authoritarian leadership may weaken it (10, 47). National and organizational culture may also play a role, as collectivist cultures may place greater emphasis on trust in hierarchical relationships (48).

### 2.5 Relationship between psychological safety and workplace flourishing

While the importance of psychological safety in shaping the work environment is gaining recognition, the empirical evidence is mixed. Goasi, Jowett, and Nascimento-Junior (16) found that psychological safety significantly impacted athletes’ flourishing, with psychological safety having a stronger predictive effect than relationship quality. Similarly, van der Walt and Lezar (49) and Fransen et al. (50) emphasized how flourishing within digital workplaces and sports environments can prevent burnout and foster healthy functioning. The positive social dynamics created by psychological safety and strong relationships contribute to individuals thriving in their roles.

However, other studies have found more modest or contingent effects. Zhang and Song (51) found that the relationship between psychological safety and well-being was moderated by error management climate, with stronger effects in organizations that encouraged learning from mistakes. Sharifirad (52) found that while psychological safety was positively associated with job satisfaction and lower work-related stress, these effects were weaker in organizations with high power distance. These contradictory findings suggest that psychological safety’s relationship with flourishing may depend on contextual factors such as organizational culture, leadership style, and national culture.

### 2.6 Relationship between trust and workplace flourishing

Empirical studies have established a direct impact of perceived trust on the subjective well-being of individuals (53, 54). When individuals perceive trust in their leaders and the organization, it contributes positively to their overall well-being, underscoring the broader impact of trust on the subjective experiences of employees. This suggests that a sense of security, support, and guidance provided by trusted leaders significantly influences employees’ well-being, both individually and within the organizational context. Thus, trust fosters a sense of security, support, and well-being that enables workplace flourishing.

However, the strength of this relationship may vary across contexts. Jaškevičiūtė (55) found a positive association between trust in the organization and employee well-being, but noted that this relationship was weaker in organizations with high levels of job insecurity. Dirks and Ferrin (11) identified that the negative effects of mistrust on well-being were more pronounced in organizations with low procedural justice. These context-dependent findings highlight the need to examine trust-wellbeing relationships in specific organizational settings. Conversely, Dirks and Ferrin (11) point out the adverse effects of mistrust, such as increased anxiety and its subsequent negative influence on well-being.

### 2.7 Critical synthesis and theoretical integration

While the independent associations between trust, psychological safety, and flourishing are well-documented, the literature reveals several unresolved theoretical questions that this study addresses.

First, the directionality of the trust-psychological safety relationship remains unclear. Basit (13) and Li and Yan (15) suggest that trust enables psychological safety, while Mitterer and Mitterer (14) argue that psychological safety enables trust. The literature has not systematically tested both directions within a single study, particularly in hierarchical contexts. This study addresses this gap by testing competing serial mediation paths: Trust → PSS → WPF versus PSS → Trust → WPF.

Second, most studies have been conducted in Western or Asian civilian contexts (e.g., 16–18). The extent to which these relationships hold in hierarchical, high-stakes, and Sub-Saharan African military contexts remains unknown. This study extends the literature by examining these relationships in the Ghana Armed Forces.

Third, contradictory findings in the literature—ranging from strong positive associations to weak or non-significant effects—suggest that moderators and boundary conditions are under-explored. Studies by Maximo et al. (56) found no significant mediation of trust in the authentic leadership-psychological safety relationship, while Zhang and Song (51) found that the psychological safety-well-being relationship was moderated by error management climate. These contradictory findings highlight the need for context-specific investigations, particularly in hierarchical military settings where trust dynamics may operate differently.

Fourth, while psychological safety has been studied extensively at the team level, less is known about how individual perceptions of psychological safety relate to flourishing in hierarchical contexts where team-level climate may be differentially experienced based on rank and position. This study contributes by examining individual-level perceptions of psychological safety in a military context.

### 2.8 Summary and hypotheses

Based on the literature reviewed and the critical synthesis, the following hypotheses were tested:

H1 Psychological safety is positively associated with workplace flourishing.
H2 Trust in leadership is positively associated with workplace flourishing.
H3 Trust in leadership is positively associated with psychological safety.
H4a Trust in leadership is indirectly associated with workplace flourishing through psychological safety (serial mediation: Trust → Psychological Safety → Workplace Flourishing).
H4b Psychological safety is indirectly associated with workplace flourishing through trust in leadership (serial mediation: Psychological Safety → Trust → Workplace Flourishing).

## 3 Theoretical framework

This study employs the Job Demands-Resources (JD-R) theory (19) as its central theoretical framework to examine how trust and psychological safety relate to workplace flourishing in high-pressure occupational settings. JD-R theory is particularly appropriate for this study because it provides a comprehensive framework for understanding how job characteristics influence employee well-being and performance (19, 57). The theory posits that job characteristics can be categorized into two broad categories: job demands and job resources.

Job demands refer to aspects of a job that require sustained physical or psychological effort and are therefore associated with physiological and psychological costs (19). In military contexts, job demands include operational risks, uncertainty, time pressure, high-stakes decision-making, rigid hierarchies, and prolonged separation from family and support networks. These demands can deplete employees’ psychological resources and impede well-being if not balanced by adequate resources.

Job resources refer to aspects of a job that help employees achieve work goals, reduce job demands and the associated physiological and psychological costs, and stimulate personal growth and development (57). Job resources can be organizational (e.g., career opportunities, job security), social (e.g., supportive leadership, team cohesion), psychological (e.g., autonomy, feedback), or physical (e.g., safe working conditions).

### 3.1 The motivational process

JD-R theory posits that job resources initiate a motivational process that leads to positive outcomes such as work engagement, job satisfaction, and well-being (19, 58). When employees have access to adequate job resources, they are more likely to experience intrinsic motivation, personal growth, and positive health outcomes. This motivational process is particularly relevant in high-stress contexts where job demands are high, as resources can buffer the negative effects of demands and promote well-being (59, 60).

### 3.2 Trust and psychological safety as job resources

Within the JD-R framework, trust in leadership and psychological safety can be conceptualized as critical job resources that enable flourishing in demanding military contexts. Trust functions as a relational resource—a social support mechanism that reduces uncertainty, enhances cooperation, and enables employees to invest their cognitive and emotional energy in productive work rather than self-protection (9, 11). When employees trust their leaders, they conserve resources that would otherwise be expended on vigilance, political maneuvering, or coping with interpersonal threats. Psychological safety functions as a climate resource—a shared perception that the team environment is safe for interpersonal risk-taking (10). In high-pressure contexts where mistakes can have serious consequences, psychological safety enables the very behaviors that drive adaptation and excellence: prompt error reporting, open discussion of problems, collaborative problem-solving, and knowledge sharing. When employees feel psychologically safe, they can fully engage in their work without fear of negative consequences to their self-image, status, or career (37).

### 3.3 The sequential resource pathway: theoretical rationale

JD-R theory posits that job resources can operate sequentially—that is, certain resources enable the development of other resources (19, 61). This sequential resource accumulation is consistent with Conservation of Resources (COR) theory, which suggests that resources tend to aggregate and that the presence of one resource facilitates the acquisition of others (62).

Drawing on this principle, this paper proposes that in hierarchical military contexts, psychological safety functions as a foundational climate resource that enables the development of trust as a relational resource. This sequential logic is supported by several theoretical considerations:

1. Hierarchical Context: In military organizations, personnel operate within rigid hierarchical structures where rank and chain of command shape interactions. Before individuals can develop trust in their leaders, they may first need to perceive that the environment is psychologically safe—that they can express concerns, admit mistakes, and take interpersonal risks without fear of reprisal. Psychological safety provides the conditions under which trust can emerge.
2. Resource Conservation: Psychological safety reduces the cognitive and emotional energy that personnel would otherwise expend on self-protection and vigilance. This resource conservation frees individuals to invest in developing trusting relationships with their leaders. When personnel feel safe, they are better able to evaluate their leaders’ trustworthiness and respond with trust.
3. JD-R Theory: JD-R theory suggests that job resources operate through motivational processes. Psychological safety—as a climate resource—initiates a motivational process that enables the development of trust—as a relational resource. This sequential resource pathway is consistent with the JD-R framework’s emphasis on resource caravans and resource accumulation.

### 3.4 Deriving hypotheses from JD-R theory

**Table:** Table A. Linking hypotheses to study theory.

*Hypothesis*	JD-R justification
*H1: Psychological safety is positively associated with workplace flourishing.*	Psychological safety, as a climate resource, initiates a motivational process that leads to positive health outcomes (flourishing) (57).
*H2: Trust in leadership is positively associated with workplace flourishing.*	Trust, as a relational resource, enhances the motivational process by reducing uncertainty and enabling employees to invest their resources in productive work (11).
*H3: Trust in leadership is positively associated with psychological safety.*	Resources tend to aggregate; trust may facilitate the perception of psychological safety (61).
*H4a: Trust is indirectly associated with workplace flourishing through psychological safety.*	Trust as a relational resource may enable the development of psychological safety as a climate resource, which then contributes to flourishing. (13, 15).
*H4b: Psychological safety is indirectly associated with workplace flourishing through trust.*	Psychological safety as a climate resource enables the development of trust as a relational resource, which then contributes to flourishing (10, 14).

### 3.5 Which pathway does the theory predict?

While both directions are theoretically plausible, JD-R theory—combined with the hierarchical nature of military organizations—provides stronger support for the pathway where psychological safety enables trust (PSS → PST → WPF). This is because (a) climate resources (psychological safety) are generally considered more foundational than relational resources (trust) in JD-R theory (b) in hierarchical contexts, individuals first assess environmental safety before making relational decisions (14) (c) motivational process in JD-R theory typically flows from contextual factors to individual outcomes.

However, given the bidirectional evidence in the literature (13, 15), this paper test both pathways to determine which is more dominant in the military context. Testing both serial mediation pathways (H4a and H5b) represents the primary theoretical contribution of this study. Also, outcome has different implications for organizational interventions: if trust precedes psychological safety, leadership development should prioritize trust-building; if psychological safety precedes trust, creating safe environments should be the priority.

## 4 Methodology

This study adopted an explanatory research design which aims to examine the structural relationships among specific constructs (trust, psychological safety, and workplace flourishing). The core objective is to test theoretically derived hypotheses regarding the associations between these constructs and to assess the fit of the proposed model with the observed data. These constructs are designed to provide comprehensive explanations for the observed phenomena, facilitating the process of generalization (63–65). A quantitative design was adopted using a cross-sectional survey. Quantitative methods are adept at providing precise and succinct responses to research inquiries and at extrapolating findings to a broader population. This is typically achieved through the measurement of specific characteristics within a sample and the analysis of information collected from a well-planned and structured data collection process (66). For this research, the population consisted of military personnel, including Commissioned Officers and Non-Commissioned Officers (NCOs), serving in the Ghana Armed Forces (GAF), specifically those stationed at the 37 Military Hospital in the Greater Accra Region.

The 37 Military Hospital was selected as the study site for several strategic reasons. First, it is a tri-service facility that houses personnel from all three branches of the Ghana Armed Forces—Army, Navy, and Air Force—providing a cross-sectional representation of the broader military population within a single location. Second, the hospital’s central location in Greater Accra and its role as the primary military medical facility in Ghana ensured access to a diverse range of personnel across different ranks, occupational roles, and operational backgrounds. Third, the practical constraints of conducting research within a military context—including security considerations, operational demands, and access restrictions—made a single-site, concentrated approach more feasible while still providing a heterogeneous sample. However, this study acknowledge that the single-site design limits the generalizability of its findings to the entire Ghana Armed Forces. We position this study as providing initial empirical evidence within a specific military context, recognizing that replication across multiple military installations and units would be necessary to establish broader generalizability. The findings should be interpreted as indicative of relationships within this particular organizational setting, with future research needed to test their applicability across the wider military population.

The targeted military personnel comprised ranks ranging from Second Lieutenant (2/Lt) to Major (Maj) among Commissioned Officers, and from Warrant Officers to Privates among NCOs, including equivalents in other Arms of Service. Out of 619 total populations, a sample size of 370 personnel was determined using formula: n=N/1+N(e^2^). Stratified sampling technique was used to ensure adequate representation within each stratum —Army, Navy, and Air Force—within the GAF. In terms of inclusion and exclusion criteria, the study targeted personnel in the armed forces across various units and departments within the Forces. The inclusion criteria established for participants were as follows. First, participants had to be currently serving members of the Ghana Armed Forces to ensure relevance to the study context. Second, participants were required to be actively engaged in their military responsibilities within the Ghana Armed Forces during the time of data collection to capture their current experiences. Third, the study included participants from the ranks of Second Lieutenant to Major (commissioned officers) and Non-Commissioned Officers (NCOs), as well as their equivalents in other branches of service. The exclusion criteria established for the study were as follows. First, senior officers, defined as those holding ranks from Lieutenant Colonel and above, including Generals, Brigadier Generals, Commodores, Admirals, Colonels, Marshals of the Air Force, and Air Commodores, as well as their equivalents in other services, were excluded from participation. From a theoretical rationale, **t**his study focuses on how military personnel perceive trust in leadership, psychological safety, and their relationship with workplace flourishing. The most appropriate perspective for examining these relationships is that of personnel who are primarily in follower or recipient roles of leadership—those who receive guidance, direction, and influence from their superiors. Senior officers, by virtue of their rank and position, are primarily responsible for exercising leadership rather than receiving it in the same capacity as junior personnel. Their experiences of trust and psychological safety may differ systematically from those in follower roles due to their decision-making authority, autonomy, and different interactions with superiors. Including senior officers could introduce variance that is not representative of the broader personnel experience and could conflate the relationships under investigation. Second trust in leadership and psychological safety may operate differently across hierarchical levels. Senior officers have different levels of autonomy, different decision-making authority, and different patterns of interaction with superiors compared to junior personnel. By focusing on personnel up to the rank of Major, the study captures the perspectives of those who are still primarily in follower roles while maintaining sufficient hierarchical diversity. Lastly from a practical standpoint, Senior officers have demanding schedules, limited availability, and restricted accessibility due to their operational and administrative responsibilities. Their inclusion would have significantly increased the logistical complexity of data collection and may have reduced the overall response rate. The practical constraints of conducting research in a military setting necessitated a focused approach.

Second, non-military personnel, including civilians or individuals not directly affiliated with the Ghana Armed Forces, were also excluded. This decision aimed to maintain the homogeneity of the sample and ensure the unique military context was preserved, thereby facilitating the attainment of the research objectives.

### 4.1 Measures

Standardized questionnaires were adopted to measure each construct. Workplace flourishing scale measures the overall sense of well-being at the workplace which was assessed using the Flourishing at work scale - FAWS adopted from Rothmann, (22) and used by Rautenbach (29). The 5-point response format from “never, once or twice, unsure, few times and everyday”). It is one of the most extensively used and tested FAW measures with high convergent and discriminant validity results. After CFA, 5 items were retained for the final analysis. Trust in leader scale was developed by Adams and Sartori Adams, and McCann (67). Participants evaluated these items using a seven-point Likert-type scale, ranging from 1 (completely disagree) to 7 (completely agree). Some of the statements include, “*I know my leader will keep their word, I can rely on my leader to behave predictably*”. After CFA, 4 items were retained for the final analysis. The Psychological Safety Scale developed by Edmondson (10) was adopted to measure the variable. It is made up of 7 items with a 7 Likert response ranging from 1=very inaccurate to 7=very accurate). Some of the items include “*Members of this team can bring up problems and tough issues; No one on this team would deliberately act in a way that undermines my efforts; Working with members of this team, my unique skills and talents are valued and utilized*”. After CFA, 4 items were retained for the final analysis.

### 4.2 Response format consideration

Worth indicating that the study employed varied response formats across constructs to reduce the risk of cognitive consistency bias, which can inflate observed relationships when uniform response formats are used (68). Workplace flourishing was assessed using a 5-point frequency scale ranging from “never” to “every day,” while trust in leadership and psychological safety were measured using 7-point Likert-type agreement scales. This variation in response formats is a procedural remedy for common method bias, as it reduces the likelihood of participants responding mechanically across all items. On the other hand, while Edmondson’s scale was originally designed to measure team-level shared beliefs, the scale has been widely validated and used at the individual level of analysis in numerous organizational studies (e.g., 12, 41, 43). Individual-level analysis is appropriate for this study for several reasons. First, we are interested in how individual military personnel perceive their psychological safety in relation to their leaders, as these perceptions may vary even within the same team due to differences in rank, experience, or personal relationships. Second, given the hierarchical nature of military organizations, individuals may experience psychological safety differently based on their position in the chain of command. Third, the use of mixed response formats within a single SEM model is methodologically appropriate. SEM with Maximum Likelihood (ML) estimation is robust to variations in scale formats when data are treated as continuous and when the number of response categories is sufficient (five or more categories is generally considered acceptable; 69). Given that all constructs in this study have at least five response categories, the mixed formats do not pose a threat to the validity of the SEM result.

### 4.3 Reliability and validity of measures

All scales used in this study have demonstrated strong psychometric properties in previous research. Cronbach’s alpha coefficients from prior studies were as follows: Flourishing at Work Scale (α = .950; 22, 29); Trust in Leader Scale (α = .948; 67); and Psychological Safety Scale (α = .720; 10). Following data collection, reliability and validity were reassessed in the current sample using Confirmatory Factor Analysis (CFA). The results, presented in **Table 2**, confirmed acceptable reliability and validity for all constructs.

### 4.4 Adaptation for the Ghanaian military context

All scales were administered in English, the official language of the Ghana Armed Forces. To ensure relevance and appropriateness for the military context, the questionnaire was pre-tested with a sample of military personnel (n = 15) representing the target population—including both commissioned officers and non-commissioned officers from the Army, Navy, and Air Force. The pre-test assessed the clarity, comprehensibility, and face validity of all items within the military context. Minor modifications were made to ensure contextual relevance while preserving the psychometric integrity of the established scales. The questionnaire was also reviewed by academic supervisors and military liaison officers familiar with the Ghana Armed Forces context.

This study was conducted in strict accordance with ethical principles for research involving human participants. Before data collection commenced, ethical approval was obtained from two independent bodies: the Ethics Committee for Humanities at the University of Ghana (March 2024) and the Institutional Review Board (IRB) of the 37 Military Hospital (May 2024).

### 4.5 Survey administration

The survey was administered using a mixed-mode approach to accommodate participants’ availability and preferences. Paper-based questionnaires were distributed in person to military personnel across various wards, departments, and training units within the 37 Military Hospital. To ensure accessibility for personnel with demanding schedules, particularly senior officers and those on operational duties, an online version was also created using Google Forms. Questionnaires were completed voluntarily and independently by participants. While focal point persons assisted with questionnaire distribution and collection, participants were not supervised during completion to ensure candor. To further encourage honest responses, participants were assured of the confidentiality and anonymity of their responses; they were instructed not to write their names or any identifying information on the survey. Detailed instructions emphasized that there were no right or wrong answers and that honest responses were valued. Participants were informed that their responses would not be shared with their commanders or used for performance evaluation purposes. This approach was designed to reduce social desirability pressures, which are particularly relevant in a military hierarchy where respondents may hesitate to provide negative evaluations of leadership.

Given the exclusive reliance on self-report measures collected at a single point in time, we assessed the potential influence of common method bias using multiple approaches (68, 70). First, a Harman’s single-factor test was conducted to determine whether a single latent factor accounts for the majority of covariance among the measures (71). All items measuring the three focal constructs—trust in leadership (4 items retained after CFA), psychological safety (4 items), and workplace ace flourishing (5 items)—were entered into an exploratory factor analysis using principal axis factoring with no rotation. The unrotated factor solution was examined to identify the number of factors emerging and the variance explained by each factor. The analysis extracted three factors with eigenvalues greater than 1.0, which together accounted for 62.4% of the total variance. Critically, the first factor accounted for only 34.7% of the variance, well below the 50% threshold that would indicate a substantial common method bias problem (68). The remaining factors accounted for 17.2% and 10.5% of the variance, respectively. The presence of multiple factors and the absence of a single dominant factor suggest that common method bias does not pose a significant threat to the validity of our findings.

Second, implemented procedural remedies during data collection to minimize the risk of common method bias, including:

- the anonymity of responses was strongly emphasized, and participants were informed that their responses would not be shared with their commanders. Second, the survey was administered with the assistance of focal point persons who were not part of the participants’ direct chain of command, reducing perceived risks of reprisal.
- Using varied response formats (frequency-based scales for workplace flourishing, 7-point Likert scales for trust and psychological safety) to reduce cognitive consistency biases;
- Providing clear instructions emphasizing that there were no right or wrong answers; and
- Pre-testing the questionnaire with military personnel (n=15) to ensure item clarity and contextual relevance. Collectively, the statistical tests and procedural measures indicate that common method bias is unlikely to have substantively influenced the findings of this study.

Also of the 370 questionnaires distributed, 355 were returned. Following data cleaning, 63 responses were excluded due to incomplete data (missing values exceeding 10% per questionnaire), resulting in a final usable sample of 292 responses. This represents a response rate of 78.9% (292/370) for the usable sample and 82.2% (355/370) for returned questionnaires. Missing values were addressed using the Expectation-Maximization (EM) method (72, 73), which generates robust estimators that account for missing data regardless of sample size or the amount of missing data. The number of missing values in the dataset was less than 10% of the total data points. To assess potential non-response bias, we compared early and late respondents on key demographic characteristics (age, gender, rank, and branch of service) following the recommendations of Armstrong and Overton (74). Independent samples t-tests and chi-square tests revealed no significant differences between early and late respondents on any demographic variable (all p >.05), suggesting that non-response bias is unlikely to have substantially influenced the findings.

To protect participant privacy, all data were collected and handled with strict confidentiality. No personally identifiable information was recorded on the questionnaires. All data were stored securely and were accessible only to the research team, with all identifying information scheduled for deletion after the study’s completion. In terms of analysis, Structural Equation Modeling (SEM) was used.

## 5 Findings and discussion

### 5.1 Descriptive statistics -*demographics*

The analysis revealed that 57% of respondents were between 30 to 39 years, 30% were between 18 to29 years, and 14% were between 40 to 49 years. In terms of gender, 66% of the respondents were male, while 34% were female, reflecting the gender composition of the workforce. The type of force respondents belonged to was also analyzed, with 47% from the Army, and 26% each from the Navy and Air Force (kindly view above **Table A**).

**Table 1:** Summary of demographic characteristics.

Characteristic	Category	Frequency	Percentage (%)
Age	18–29 years	87	29.8
	30–39 years	165	56.5
	40–49 years	40	13.7
Gender	Male	191	65.5
	Female	101	34.6
Marital Status	Married	83	28.4
	Single	182	62.3
	Divorce/widowed	27	9.2
Educational Level	SSS/SHS	61	20.9
	Voc./Tech	45	15.4
	Tertiary	147	50.3
	Post-Graduate	39	13.4
Work Experience	Less than a year	15	5.1
	1–5 years	133	45.5
	6–10 years	98	33.6
	11–15 years	33	11.3
	16–20 years	12	4.1
	21 years and above	1	.3
Type of Force	Army	138	47.3
	Navy	77	26.4
	Air force	77	26.4
Total		292	100

Source: Field Data (2024)

### 5.2 Descriptive statistics and correlations

**Table 2** presents the descriptive statistics, correlations, and reliability coefficients for the study variables. The analysis revealed significant positive correlations between trust in leadership and workplace flourishing (r = .347, p <.01), psychological safety and workplace flourishing (r = .242, p <.01), and trust in leadership and psychological safety (r = .289, p <.01). All Cronbach’s alpha coefficients exceeded the recommended threshold of.70 (75), ranging from.711 to.912, indicating good internal consistency. Composite Reliability (CR) values ranged from.747 to.918, and Average Variance Extracted (AVE) values ranged from.499 to.737, meeting or exceeding recommended thresholds (76, 77).

**Table 2:** Descriptive statistics, correlations, and reliability coefficients.

Variable	Mean	SD	1	2	3	Cronbach’s α	CR	AVE
1. Trust in Leadership	4.567	.633	**(.859)**			.912	.918	.737
2. Psychological Safety	4.416	.582	.289**	**(.820)**		.886	.889	.672
3. Workplace Flourishing	5.607	.942	.347**	.242**	**(.707)**	.711	.747	.499

N = 292. *p <.01. Square root of AVE shown in bold on diagonal. SD, Standard Deviation; CR, Composite Reliability; AVE, Average Variance Extracted.

### 5.3 Assessment of common method bias

Given the exclusive reliance on self-report measures collected at a single point in time, we assessed the potential influence of common method bias using Harman’s single-factor test (68, 71, 78). All items measuring the three focal constructs—trust in leadership (4 items retained after CFA), psychological safety (4 items), and workplace flourishing (5 items)—were entered into an exploratory factor analysis using principal axis factoring with no rotation. The analysis extracted three factors with eigenvalues greater than 1.0, which together accounted for 62.4% of the total variance. Critically, the first factor accounted for only 34.7% of the variance, well below the 50% threshold that would indicate a substantial common method bias problem (68). The presence of multiple factors and the absence of a single dominant factor suggest that common method bias does not pose a significant threat to the validity of this study findings.

Second, procedural remedies were implemented during data collection to minimize the risk of common method bias (70), including ensuring respondent anonymity, using varied response formats across constructs, providing clear instructions emphasizing the absence of right or wrong answers, and pre-testing the questionnaire with military personnel (n=15) to ensure item clarity and contextual relevance. Collectively, these procedural and statistical measures indicate that common method bias is unlikely to have substantively influenced the findings of this study.

### 5.4 Multicollinearity assessment

To assess potential multicollinearity among predictor variables, Variance Inflation Factors (VIF) were calculated. VIF values for trust in leadership and psychological safety were 1.09 and 1.09, respectively. All VIF values were well below the recommended threshold of 10 (and the more conservative threshold of 5), indicating that multicollinearity does not pose a concern in this study (77). This confirms that the predictor variables are sufficiently independent, allowing for reliable estimation of the structural relationships. Subsequently, the structural model was tested to examine the direct associations between trust in leadership, psychological safety, and workplace flourishing.

### 5.5 Analytical strategy: structural equation modeling

To test the hypothesized model positing the effects of trust in leadership and psychological safety on workplace flourishing, this study employed Structural Equation Modeling (SEM) using Amos (Version 21) (79). SEM was selected for its ability to simultaneously assess the measurement model (validity of constructs) and the structural model (relationships between constructs), providing a robust test of the proposed association. The analysis followed a two-step procedure.

First, a Confirmatory Factor Analysis (CFA) was conducted to evaluate the validity of the three latent constructs. The measurement model demonstrated acceptable fit to the data: χ²/df = 2.375, Comparative Fit Index (CFI) = .910, Incremental Fit Index (IFI) = .911, Root Mean Square Error of Approximation (RMSEA) = .069, and Standardized Root Mean Square Residual (SRMR) = .061. All factor loadings were significant (p <.001) and exceeded the recommended threshold of.60 (77) confirming the constructs were well-defined by their respective indicators.

The Average Variance Extracted (AVE) for each construct met or exceeded.50, and the square root of the AVE for each construct was greater than its correlations with other constructs, establishing adequate convergent and discriminant validity (76, 77). Composite Reliability (CR) values ranged from.747 to.918, exceeding the recommended threshold of.70 (80), indicating good internal consistency. Cronbach’s alpha coefficients ranged from.711 to.912, further confirming the reliability of the scales.

The structural model exhibited a good fit with the data (χ²/df = 2.406, GFI = .973, CFI = .936, IFI = .940, RMSEA = .070, SRMR = .075, Pclose = .122). The model accounted for approximately 19.6% of the variance in workplace flourishing (R² = .196), indicating a modest but meaningful proportion of explained variance. This suggests that while trust in leadership and psychological safety are significant correlates of workplace flourishing, other factors not captured in this model also contribute to flourishing in this military context.

The path coefficients were examined to test the study’s hypotheses. H1 predicted that psychological safety would have a positive direct association with workplace flourishing (refer to **Table 3**). This hypothesis was supported. The SEM analysis revealed a statistically significant positive path coefficient (β = 0.111, SE = 0.034, p <.05). This indicates that for every unit increase in psychological safety, workplace flourishing increases by 0.111 units, confirming that a climate of interpersonal risk-taking is positively associated with employee well-being. The significant association implies that personnel who feel psychologically safe are more likely to flourish, as they feel supported in expressing their ideas and concerns. When team members feel that mistakes are treated as learning opportunities rather than being held against them, it fosters a supportive atmosphere. This sense of safety allows individuals to discuss challenges openly, contributing to trust and belonging within the team, which in turn is associated with greater job satisfaction and excitement in their roles. Hypothesis 1 was accepted.

**Table 3:** Direct model pathway assessment.

Hypothesis	Predictor	Predicted	Estimate	S.E	C.R	P-value	Interpretation
H1	PSS	→ WPF	.111	.034	2.013	.044	Significant
H2	PST	→ WPF	.296	.031	5.550	.001	Significant
H3	PST	→PSS	.341	.048	5.670	.001	Significant
	PSS	→PST	.264	.052	4.678	.001	Significant

Field Data (2024); Note: WPF, Workplace flourishing; PSS, Psychological Safety; PST, Trust in leadership.

H2 predicted that trust in leadership would have a positive direct association with workplace flourishing. This hypothesis was also strongly supported. The analysis found a significant and more substantial positive relationship (β = 0.296, SE = 0.031, p <.001). This result reveals trust as having a stronger statistical association with flourishing in this context, suggesting that employees’ confidence in their leaders’ integrity and reliability may be particularly important for their holistic well-being. The final model, with both predictors included, explains a significant portion of the variance in workplace flourishing. The results affirm that both trust in leadership and psychological safety are significant correlates of workplace flourishing within the Ghana Armed Forces, with trust demonstrating a comparatively stronger direct association.

H3 predicted that trust in leadership would have a positive direct association with psychological safety. The analysis found a significant positive relationship (β = 0.341, SE = 0.048, p <.001). The hypothesis was supported.

### 5.6 Effect sizes and practical significance

Beyond statistical significance, it is important to consider the magnitude of the observed associations. The model explained approximately 19.6% of the variance in workplace flourishing (R² = .196), indicating a modest but meaningful proportion of explained variance. This suggests that while trust in leadership and psychological safety are significant correlates of workplace flourishing, other factors not captured in this model also contribute to flourishing in this military context.

The association between trust in leadership and workplace flourishing (β = .296) was substantially stronger than the association between psychological safety and workplace flourishing (β = .111). This difference in effect sizes suggests that in hierarchical military contexts, trust in leadership may play a particularly important role in relation to employee flourishing. The relatively modest effect size for psychological safety (β = .111) warrants cautious interpretation. While statistically significant (p <.05), the practical significance of this finding should be considered in light of the military context, where hierarchical structures, command relationships, and operational demands may limit the extent to which psychological safety manifests and influences individual well-being.

### 5.7 Serial mediation analysis

Serial mediation was tested using bootstrapped indirect effects with 5000 resamples to obtain 95% confidence intervals (81).

H4a predicted that trust in leadership would be indirectly associated with workplace flourishing through psychological safety (Trust → Psychological Safety → Workplace Flourishing). The analysis output in **Table 4** revealed that this serial mediation path was not significant (Indirect Effect = .0379, 95% CI [.0085,.0712]). While the confidence interval does not include zero, the indirect effect was weak and did not reach the threshold for meaningful serial mediation in this military context. The hypothesis was rejected. Several factors may explain this result (a)First, in military organizations, personnel may first need to feel that the environment is psychologically safe before they can fully develop trust in their leaders. The hierarchical nature of military institutions may mean that psychological safety serves as a foundation upon which trust is built, rather than trust serving as a foundation for psychological safety. (b)Second, military structures emphasize rank and chain of command. In such environments, trust may be influenced more by one’s position in the hierarchy and perceptions of the overall climate than by the reverse process. Personnel may first assess whether the environment is safe for expression before deciding whether their leaders can be trusted.

**Table 4:** Summary of serial mediation analysis.

Hypothesis	Serial Path	Indirect Effect	SE	95% CI (LLCI)	95% CI (ULCI)	Result
H4	PST → PSS → WPF	.0379	.0156	.0085	.0712	Rejected
Alternative(H4b)	PSS → PST → WPF	.0781	.0206	.0137	.0939	Supported

Source: Field Data (2024)

**Figure 1:**
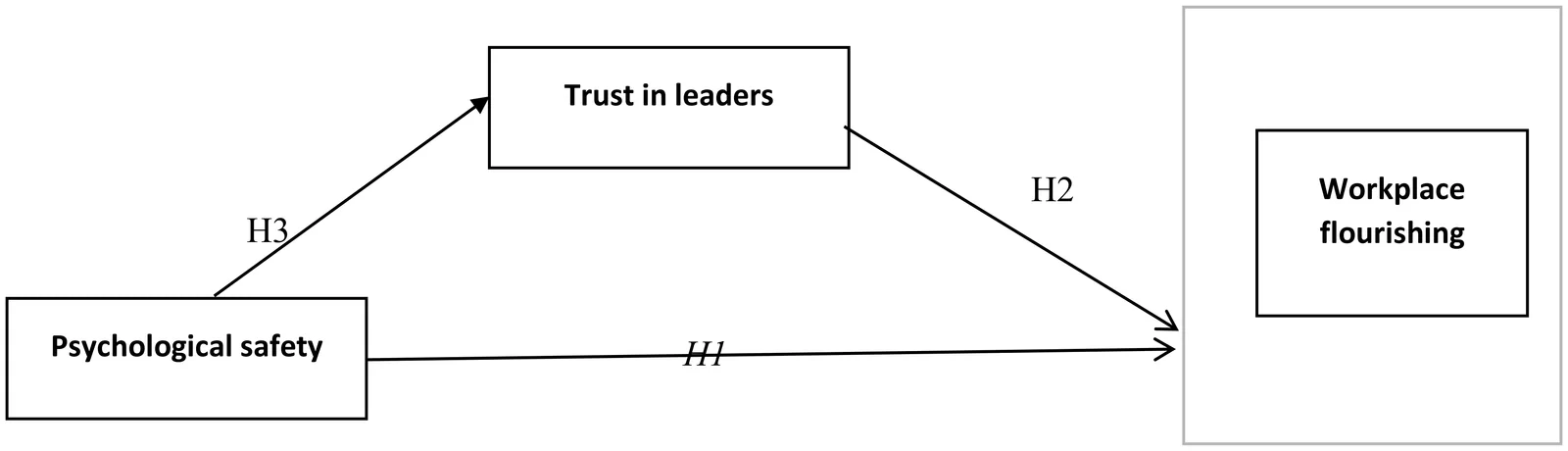
Conceptual framework.

In terms of alternative finding(H4b), the analysis revealed a significant serial mediation effect where psychological safety is indirectly associated with workplace flourishing through trust in leadership (Psychological Safety → Trust → Workplace Flourishing; Indirect Effect = .0781, 95% CI [.0137,.0939]). This finding indicates that psychological safety’s association with workplace flourishing operates through trust in leadership. Personnel who feel psychologically safe are better able to develop trust in their leaders, and this trust, in turn, is associated with higher levels of workplace flourishing. The findings as displayed in **Figure 1** indicated that psychological safety's association with flourishing through trust, rather than the reverse, may be explained by the hierarchical nature of military organizations. In such contexts, personnel may first need to perceive that the environment is psychologically safe—that they can express concerns without fear of reprisal—before they can develop trust in their leaders. This is consistent with Mitterer and Mitterer’s (14) argument that “when employees feel safe, they are more likely to build trusting relationships”.

The rejection of H4a and support for H4b has important theoretical implications. It suggests that in hierarchical military contexts, psychological safety is the foundation upon which trust is built, not the reverse. This extends the JD-R framework by demonstrating that climate resources (psychological safety) enable the development of relational resources (trust), which in turn contribute to positive outcomes (flourishing). The findings also contribute to the broader literature on trust and psychological safety by clarifying the directionality of their relationship in a hierarchical, high-stakes context.

## 6 Discussion

This study examined the pathways to workplace flourishing by investigating the roles of trust in leadership and psychological safety in a high-pressure military environment. The findings reveal a significant serial mediation pathway where psychological safety is indirectly associated with flourishing through trust. This suggests that in hierarchical military contexts, psychological safety serves as a foundation that enables trust to develop, ultimately contributing to flourishing.

### 6.1 Trust and workplace flourishing

The relationship between trust and workplace flourishing was found to be significant and substantial. The considerable path coefficient (β = 0.296) suggests that trust plays a critical role in fostering an environment associated with flourishing. Such a robust figure can be interpreted as a reflection of the importance of trust in military settings, where cohesion and morale are paramount. In high-pressure environments, like those often found in military operations, trust may act as a buffer against stress and uncertainty. When personnel trust their leaders, they are likely to experience increased emotional and psychological well-being, thereby enhancing workplace flourishing. This finding aligns closely with existing literature that emphasizes the critical role of trust in fostering employee well-being (55, 82–84). Conversely, the literature also notes the detrimental effects of mistrust, as highlighted by Dirks and Ferrin (11). Their findings regarding the adverse impacts of mistrust, such as heightened anxiety, add depth to our understanding of the dynamics at play. The current study highlights that trust has a positive effect on workplace flourishing. However, it is also crucial to recognize that a lack of trust can have serious negative effects. This shows that while trust can boost well-being and productivity, mistrust can harm individuals and the overall work environment.

### 6.2 Psychological safety and workplace flourishing

The relationship between psychological safety and workplace flourishing was also significant, though more modest (β = 0.111, SE = 0.034, p <.05). The relatively modest association warrants careful consideration. Several factors may explain this finding. First, the hierarchical and command-driven nature of military organizations may limit the extent to which psychological safety manifests and influences individual well-being. In environments where orders are expected to be followed without question and rank dictates communication patterns, the felt permission to speak up or take interpersonal risks may be constrained, reducing its potential impact on flourishing. Second, psychological safety may be experienced differently across ranks. Junior personnel may have fewer opportunities to voice concerns or influence decisions compared to officers, potentially attenuating the overall association with flourishing. Third, the military’s emphasis on resilience, discipline, and mission focus may mean that personnel derive well-being from other sources—such as camaraderie, sense of purpose, or organizational identification—that are not captured by psychological safety. Although the effect size for psychological safety was more modest compared to trust, it remains statistically significant. This suggests that psychological safety contributes to a supportive environment where personnel can express their ideas and concerns without fear of repercussions. This sense of safety is crucial in high-stakes military settings, where open communication can lead to improved decision-making and operational effectiveness. The relatively modest effect size may also reflect the military’s hierarchical structure, where individuals may experience psychological safety differently based on rank, command relationships, or operational context.

While fewer studies have directly examined this connection, recent research by Goasi, Jowett, and Nascimento-Junior (16) highlights the importance of transformational leadership in fostering psychological safety and, consequently, flourishing. This aligns with the study, indicating that leaders who create psychologically safe environments enable personnel to express themselves, ultimately enhancing their well-being. Furthermore, Goasi et al. emphasize the mediating roles of relationship quality and psychological safety in promoting flourishing, suggesting that a supportive environment is crucial for thriving. This echoes the findings from van der Walt and Lezar (49) and Fransen et al. (50), which highlight the significance of flourishing in various contexts, including sports and digital workplaces. The emphasis on the role of psychological safety in preventing burnout and promoting positive outcomes reinforces the argument that this variable is critical in facilitating workplace flourishing.

### 6.3 Comparing the associations of trust and psychological safety

The substantially stronger association between trust in leadership and workplace flourishing (β = .296) compared to psychological safety (β = .111) warrants attention. This difference may reflect the hierarchical nature of military organizations, where trust in leadership is particularly salient. In command-driven environments, personnel rely heavily on their leaders for direction, protection, and welfare. When trust is present, it may reduce uncertainty and create a sense of security that is foundational to well-being. Also, the stronger association of trust may reflect that trust operates at multiple levels—interpersonal, organizational, and systemic—whereas psychological safety may be more localized to immediate team dynamics. This multi-level nature of trust may amplify its association with flourishing compared to the more narrowly defined construct of psychological safety. Psychological safety, while important, may be more contingent on team-level interactions and may be experienced differently across ranks, potentially limiting its overall association with flourishing. It is important to emphasize, however, that the cross-sectional nature of our data precludes conclusions about causal primacy. The stronger coefficient for trust does not establish that trust is more “foundational” or causally prior to psychological safety. Rather, it indicates that trust shows a stronger statistical association in this sample, which may reflect contextual factors unique to the military setting.

## 7 Implications, summary, limitations, and recommendations for future research

### 7.1 Implications for mental health in military populations

The findings of this study carry important implications for the mental health of military personnel. Workplace flourishing—characterized by high levels of emotional, psychological, and social well-being—is not merely a desirable state but a protective factor against adverse mental health outcomes. In military populations, where personnel are exposed to high levels of operational stress, trauma, and prolonged separation from support networks, the absence of flourishing may manifest as burnout, psychological distress, anxiety, depression, or post-traumatic stress symptoms (21, 84). Research has consistently shown that individuals who flourish exhibit greater resilience, better coping mechanisms, and lower rates of mental health disorders compared to those who languish (30, 85).

The significant associations between trust in leadership, psychological safety, and flourishing observed in this study suggest that organizational factors—particularly the quality of leadership and the climate of interpersonal safety—may play a crucial role in supporting the mental health of military personnel. When service members trust their leaders and feel psychologically safe to voice concerns without fear of reprisal, they may be more likely to seek help for mental health challenges, report distress early, and engage in supportive peer networks (10, 12). Conversely, environments characterized by low trust and psychological safety may discourage help-seeking, normalize distress, and exacerbate the stigma associated with mental health care in military settings (86, 87).

These findings underscore the need for military organizations to view leadership development and psychological safety not only as performance-enhancing strategies but as essential components of a comprehensive mental health promotion framework. By fostering trust and psychological safety, military leaders can create conditions that support flourishing and, in turn, contribute to the prevention of burnout, early identification of distress, and improved mental health outcomes among personnel. Future research should explore whether interventions designed to enhance trust and psychological safety directly reduce mental health burden and improve help-seeking behaviors in military populations.

### 7.2 Theoretical contributions

From a theoretical perspective, the findings of this study, which establish trust and psychological safety as significant correlates of workplace flourishing, can be powerfully interpreted through the lens of the Job Demands-Resources (JD-R) theory. This framework posits that workplace well-being is a function of the balance between job demands, which are aspects of the job that require sustained effort and incur psychological costs, and job resources, which are functional in achieving goals and stimulating growth. Within this paradigm, the high-pressure military environment represents a context of intense job demands, including operational risks, uncertainty, and rigid hierarchies, which inherently threaten well-being and can inhibit flourishing.

The results demonstrate that trust in leadership and psychological safety function as critical job resources that activate a motivational pathway to flourishing. Importantly, the serial mediation finding reveals the sequential nature of these resources: psychological safety serves as a foundational climate resource that enables the development of trust as a relational resource, which in turn contributes to flourishing. In hierarchical military contexts, personnel first need to perceive that the environment is psychologically safe—that they can express concerns without fear of reprisal—before they can develop trust in their leaders. This sequential logic is consistent with JD-R theory’s emphasis on resource caravans, where the presence of one resource facilitates the acquisition of another (19, 61).

Psychological safety, as a climate resource, directly enables the “functioning well” aspect of flourishing by facilitating learning, collaboration, and authentic self-expression. When personnel feel psychologically safe, they conserve cognitive and emotional energy that would otherwise be expended on self-protection and vigilance. This resource conservation enables them to invest in developing trusting relationships with their leaders—who, in turn, provide the security and predictability that further reduce the psychological costs of job demands. Trust, as a relational resource, provides the interpersonal security that enables personnel to fully engage in their work, freeing cognitive and emotional energy for productive and meaningful engagement.

Together, these resources create a buffer against the depleting effects of job demands while simultaneously fostering the conditions for employees to invest their full selves into their work, leading to the positive health outcome identified in the JD-R model: workplace flourishing, characterized by high levels of energy, dedication, and positive relationships. The significant serial mediation pathway—Psychological Safety → Trust → Workplace Flourishing—provides empirical support for this sequential resource logic.

This theoretical narrative explains the significant positive relationships observed and positions trust and psychological safety not as peripheral attributes but as essential, functional resources that transform a depleting work environment into one that is psychologically nourishing and conducive to growth. Critically, the rejection of the reverse serial pathway (Trust → Psychological Safety → Workplace Flourishing) suggests that in hierarchical military contexts, psychological safety serves as the foundation upon which trust is built, rather than the reverse. This extends JD-R theory by clarifying the directional relationship between climate and relational resources in high-stakes, hierarchical settings. Thus, the primary contribution is the extension of existing theoretical frameworks to a new context—demonstrating the sequential resource pathway (PSS → PST → WPF) in a military setting.

Beyond the Job Demands-Resources (JD-R) framework, the findings of this study can be understood through complementary theoretical lenses. Social Exchange Theory (SET; 88) offers a valuable framework for understanding the strong association between trust in leadership and flourishing. SET posits that social interactions are governed by norms of reciprocity—individuals respond to positive treatment from others with positive attitudes and behaviors. The stronger association of trust compared to psychological safety may reflect the centrality of the leader-subordinate exchange relationship in military contexts, where hierarchical structures amplify the importance of leader behaviors.

Conservation of Resources (COR) theory (62) provides an additional lens for interpreting these findings. COR theory posits that individuals strive to obtain, retain, and protect valued resources, and that resource loss or threat of loss leads to stress. Trust in leadership and psychological safety can be conceptualized as valuable psychological resources that protect against the depletion of emotional and cognitive resources in high-stress military environments. When personnel trust their leaders and feel psychologically safe, they may be better able to conserve resources that would otherwise be expended on self-protection, vigilance, or coping with interpersonal threats. This resource conservation, in turn, supports flourishing by enabling personnel to invest their energy in positive engagement and growth rather than defensive behaviors.

## 8 Conclusion

This study examined the associations between trust in leadership, psychological safety, and workplace flourishing within the high-stakes, hierarchical environment of the Ghana Armed Forces. The findings provide empirical evidence that both trust in leadership and psychological safety are significantly associated with workplace flourishing, with trust demonstrating a stronger statistical association (β = .296) compared to psychological safety (β = .111). The model explained approximately 19.6% of the variance in workplace flourishing, with trust accounting for roughly 8-9% of unique variance and psychological safety accounting for approximately 1%.

Critically, serial mediation analysis revealed that psychological safety is indirectly associated with workplace flourishing through trust in leadership (Indirect Effect = .0781, 95% CI [.0137,.0939]). This finding suggests that in hierarchical military contexts, psychological safety may serve as a foundational climate resource that enables the development of trust as a relational resource, which in turn contributes to flourishing. The reverse serial pathway—trust leading to psychological safety leading to flourishing—was not supported, indicating that psychological safety precedes trust in this military context rather than the reverse.

These findings suggest that in military contexts, confidence in leadership may be particularly salient for well-being. The relatively modest effect of psychological safety indicates that while it contributes incrementally to flourishing, its practical significance should be interpreted with caution, particularly given the hierarchical constraints of military organizations. Importantly, the serial mediation finding suggests that psychological safety’s primary contribution to flourishing operates through enabling trust, rather than directly enhancing well-being. The cross-sectional nature of our data means that we cannot establish causality or determine whether trust and psychological safety precede flourishing, whether flourishing individuals appraise their environment more favorably, or whether these relationships are bidirectional.

### 8.1 Practical implication and recommendations

While a cross-sectional study cannot establish causality, the observed associations—particularly the serial mediation finding that psychological safety is indirectly associated with workplace flourishing through trust in leadership (PSS → PST → WPF) —offer preliminary insights that may inform practice in military and similar high-stakes organizations. The following recommendations should be viewed as practice implications consistent with correlational evidence rather than proven prescriptions. They are organized according to the sequential logic revealed by our findings: prioritizing psychological safety as a foundation for trust development.

#### 8.1.1 Priority 1: prioritize psychological safety as a foundation

The serial mediation finding (PSS → PST → WPF) suggests that in hierarchical military contexts, psychological safety serves as a foundation upon which trust is built. This implies that military organizations seeking to enhance flourishing should first prioritize creating psychologically safe environments. When personnel feel safe to express concerns without fear of reprisal, they are better able to develop trust in their leaders, which in turn is associated with flourishing. Specific actions include:

- Establishing no-fault communication protocols: Implementing structured mechanisms such as no-fault debriefs after exercises, where personnel can openly discuss mistakes and lessons learned without fear of punishment. This normalizes learning from errors and signals that the environment is safe for honest communication.
- Training leaders in psychological safety practices: Equipping leaders with skills to create psychologically safe environments—including active listening, empathetic responses to concerns, and demonstrating vulnerability by acknowledging their own mistakes. Leaders should be trained to solicit and respond constructively to subordinate input.
- Creating multiple channels for voicing concerns: Establishing both formal (e.g., confidential reporting systems, climate surveys) and informal (e.g., one-on-one check-ins, open-door policies) mechanisms for personnel to raise concerns. In hierarchical contexts, junior personnel may feel more comfortable voicing concerns in private settings rather than in group forums.

#### 8.1.2 Priority 2: build trust within a psychologically safe environment

Given the strong direct association between trust in leadership and flourishing (β = .296), trust-building remains essential. However, our findings suggest that trust-building efforts are most effective when implemented within a psychologically safe environment—where personnel already feel safe to express concerns and take interpersonal risks. Specific actions include:

- Emphasizing trust-building behaviors: Leaders should demonstrate transparency, consistency, fairness, and genuine concern for personnel welfare. These behaviors are more likely to be perceived as trustworthy when personnel already feel psychologically safe.
- Integrating trust competencies into leadership development: Leadership training programs should emphasize trust-building as a core competency, including modules on transparent communication, ethical decision-making, and demonstrating reliability. However, these programs should be preceded by or paired with training on creating psychological safety.
- Modeling trustworthiness at all levels: Senior leaders should model trust-building behaviors, as their actions set the tone for the entire organization. When senior leaders demonstrate transparency and fairness, it signals to personnel at all ranks that trust is valued and expected.

#### 8.1.3 Priority 3: monitor and evaluate organizational climate

Periodically assessing personnel perceptions of trust and psychological safety may provide useful information for organizational climate monitoring. However, assessment should be approached with caution given the correlational nature of our evidence. Specific actions include:

- Implementing confidential climate surveys: Periodic, anonymous surveys can help identify units where psychological safety or trust is low, potentially informing targeted interventions. However, such surveys should be implemented with clear communication about their purpose and safeguards to ensure anonymity and protect against unintended consequences.
- Using data to inform interventions: Survey results should be used to identify areas for targeted improvement rather than for performance evaluation of individual leaders. Unit-level feedback should be shared transparently with personnel, along with action plans for addressing identified concerns.

### 8.2 Future research directions

The recommendations above should be empirically validated through future research. Studies should employ longitudinal and experimental designs to determine whether interventions designed to enhance psychological safety and trust lead to improvements in workplace flourishing. Research should also examine (a)whether interventions translate into measurable mental health benefits for military personnel (b) whether the sequential pathway (PSS → PST → WPF) holds in other hierarchical or high-stakes contexts.

The recommendations above are offered tentatively, reflecting the correlational nature of the study evidence. Future research should employ longitudinal study to determine whether interventions designed to enhance trust and psychological safety lead to improvements in workplace flourishing. Studies should also examine whether the effects of such interventions vary across ranks, branches, or operational contexts, and whether they translate into measurable mental health benefits for military personnel.

### 8.3 Limitations and future research

The study’s findings should be interpreted in light of several methodological limitations. First, data were collected from personnel stationed at a single military hospital, which may limit the generalizability of findings to the broader Ghana Armed Forces or to military organizations in other contexts. While the tri-service composition of the hospital provides some diversity, future research should extend this investigation to multiple military installations, including operational units and headquarters, to assess whether these relationships hold across different military settings. Second, the cross-sectional design prevents any conclusions about causality or temporal ordering. The structural relationships identified in this study represent associations at a single point in time. The study recommends that future research employ longitudinal designs to examine how trust, psychological safety, and workplace flourishing evolve over time and to establish directional relationship. Likewise, examine how trust and psychological safety interact during different operational phases—from peacetime training to active deployment and post-deployment recovery. Such designs would help establish causal relationships and reveal the dynamic nature of these constructs under varying levels of stress and threat. Additionally, comparative studies across different military branches (Army, Navy, Air Force) or between military and other high-reliability organizations (such as police services, fire departments, or hospital emergency units) could illuminate the context. Also, the exclusive reliance on self-report measures presents a limitation. While procedural measures were implemented and statistical checks (Harman’s single-factor test) indicated that common method bias was not a significant concern, self-report data may still be subject to social desirability bias, particularly in a military context where respondents may hesitate to provide negative evaluations of leadership. Future research should consider incorporating objective performance measures or multi-source data (e.g., supervisor ratings, peer evaluations) to complement self-report measures. This study did not examine potential differences across ranks, branches, or years of service. Trust and psychological safety may function differently for junior personnel compared to senior officers. Future research should test for measurement invariance and moderation effects across these subgroups to provide a more understanding of these relationships. Research should also explore potential boundary conditions and negative consequences, such as investigating whether excessive psychological safety might ever compromise operational discipline or whether there are specific situations where swift, directive leadership outweighs the benefits of consensus-building. Finally, while this study focused on trust and psychological safety, other contextual and individual factors may also be relevant to workplace flourishing. Future research could explore additional mediators and moderators, such as organizational support, team dynamics, or individual personality trait.

## Data Availability

The raw data supporting the conclusions of this article will be made available by the authors, without undue reservation.
