# Correction: Understanding the pathways to workplace flourishing: the effect of trust and psychological safety in high-pressure work environments

**DOI:** 10.3389/fpsyt.2026.1985636

**Published:** 2026-09-08

**Authors:** Frontiers Production Office

**Affiliations:** Frontiers Media SA, Lausanne, Switzerland

**Keywords:** employee well-being, Ghana armed forces, hierarchical organizations, job demands-resources theory, military personnel, occupational health, psychological safety, trust in leadership

The first name of the author “Rosemond Obuobi” was misspelled in the original article. This has now been corrected.

The affiliation “Department of Management and Public Administration, Accra Technical University, Accra, Ghana” was omitted from the paper. This has now been added as affiliation 3 and assigned to Rosemond Obuobi, and subsequent affiliations have been renumbered. Rosemond Obuobi was originally assigned to affiliation 2, but this was incorrect – Accra Technical University is their only affiliation.

The original version of this article has been updated.

